# Improved automated tumor segmentation in whole-body 3D scans using multi-directional 2D projection-based priors

**DOI:** 10.1016/j.heliyon.2024.e26414

**Published:** 2024-02-15

**Authors:** Sambit Tarai, Elin Lundström, Therese Sjöholm, Hanna Jönsson, Alexander Korenyushkin, Nouman Ahmad, Mette A. Pedersen, Daniel Molin, Gunilla Enblad, Robin Strand, Håkan Ahlström, Joel Kullberg

**Affiliations:** a Department of Surgical Sciences, Uppsala University, SE-75185, Uppsala, Sweden; b Antaros Medical AB, SE-43153, Mölndal, Sweden; c Department of Nuclear Medicine & PET-Centre, Aarhus University Hospital, 8200 Aarhus N, Denmark; d Department of Biomedicine, Aarhus University, 8000 Aarhus C, Denmark; e Steno Diabetes Center Aarhus, Aarhus University Hospital, 8200 Aarhus N, Denmark; f Department of Immunology, Genetics and Pathology, Uppsala University, SE-75185 Uppsala, Sweden; g Department of Information Technology, Uppsala University, SE-75237, Uppsala, Sweden

**Keywords:** Whole-body tumor segmentation, Medical image analysis, Deep learning, Maximum intensity projection, Backprojection, Segmentation prior

## Abstract

Early cancer detection, guided by whole-body imaging, is important for the overall survival and well-being of the patients. While various computer-assisted systems have been developed to expedite and enhance cancer diagnostics and longitudinal monitoring, the detection and segmentation of tumors, especially from whole-body scans, remain challenging. To address this, we propose a novel end-to-end automated framework that first generates a tumor probability distribution map (TPDM), incorporating prior information about the tumor characteristics (e.g. size, shape, location). Subsequently, the TPDM is integrated with a state-of-the-art 3D segmentation network along with the original PET/CT or PET/MR images. This aims to produce more meaningful tumor segmentation masks compared to using the baseline 3D segmentation network alone. The proposed method was evaluated on three independent cohorts (autoPET, CAR-T, cHL) of images containing different cancer forms, obtained with different imaging modalities, and acquisition parameters and lesions annotated by different experts. The evaluation demonstrated the superiority of our proposed method over the baseline model by significant margins in terms of Dice coefficient, and lesion-wise sensitivity and precision. Many of the extremely small tumor lesions (i.e. the most difficult to segment) were missed by the baseline model but detected by the proposed model without additional false positives, resulting in clinically more relevant assessments. On average, an improvement of 0.0251 (autoPET), 0.144 (CAR-T), and 0.0528 (cHL) in overall Dice was observed. In conclusion, the proposed TPDM-based approach can be integrated with any state-of-the-art 3D UNET with potentially more accurate and robust segmentation results.

## 1 Introduction

Cancer remains one of the leading causes of mortality worldwide [1]. Early diagnosis and accurate staging are key factors for successful patient management and increased probability of survival [2]. Whole-body positron emission tomography combined with computed tomography (PET/CT), after the injection of ^18^F-fluorodeoxyglucose (^18^F-FDG), is one of the imaging techniques used for diagnostics, staging and monitoring [3,4]. PET combined with magnetic resonance imaging (PET/MRI) is another more recent, but not as widespread, tomographic imaging technique used for similar purposes [5,6]. The radiotracer ^18^F-FDG is a glucose analogue, which accumulates in regions with high glucose metabolism, e.g., malignant tumors, normal physiology, and inflammatory regions [7]. The ^18^F-FDG-avidity can vary between different cancer types and for different tumors of the same cancer type, but is generally high in cancers such as lymphoma, lung cancer, and melanoma [8]. Traditionally, lesions are detected and annotated by radiologists through visual inspection and manual delineation of axial slices (usually between 300–1000 slices for head-to-thigh or head-to-feet examinations). This visual assessment is usually time intensive, laborious, and an error prone task affected by subjective inter-rater variability [9]. Moreover, extremely small lesions may be missed by the radiologists during cancer screening, due to human error. As these lesions might grow and metastasize over time, such mistakes can be detrimental for the survival of the particular patient. Therefore, early and accurate detection of lesions is an essential task for correct treatment planning and disease free survival [10]. Imaging biomarkers such as the metabolic tumor volume (MTV) and maximum standardized uptake value (SUVmax) are associated with the severity of the cancer and important for treatment planning [[11], [12], [13]]. Hence, identification and precise segmentation of all the lesions is important and valuable to non-invasively track tumor growth of individual lesions. In addition, treatment planning for cancer requires accurate segmentation of the pathologies in order to target only the affected areas while sparing the healthy regions [14]. Automated segmentation of healthy anatomical structures such as the liver, lungs, kidneys, spleen, pancreas is a well-researched area, with state-of-the-art segmentation results typically within the range 82–98 %, depending on the organ/tissue of interest [15,16]. On the other hand, automated segmentation of tumors, e.g. in whole-body examinations, is generally a much more challenging task as they can vary significantly in terms of size, shape, texture, etc [[17], [18], [19]]. In addition, the intra-and inter-rater variability is often very high which makes it difficult to obtain standardized ground truth labels [21]. Unlike organs and tissues, always present in roughly the same location, tumors can appear in different body parts and spread throughout the body. Additionally, the difference in tumor characteristics between cancer types makes it even more challenging, especially when the lesions are very small and there is a large inter-tumor and intra-tumor heterogeneity.

The main focus of this paper is the development of a novel end-to-end framework to improve the automated detection and segmentation of ^18^F-FDG-avid lesions from whole-body PET/CT or PET/MRI scans. Despite recent advancements and encouraging results in the field of automated tumor segmentation [[18], [19], [20]], there remain some challenges that need more attention. These include the inhomogeneity in ^18^F-FDG-avidity between different cancer forms, anatomical mismatch between PET and CT or PET and MRI (often caused by motion), inter-operator variability in ground truth segmentation and uncertainty in boundary annotation of target lesions [22]. The appearance of pathological regions in PET/CT and PET/MRI is usually heterogeneous with respect to e.g. texture, shape, and location [17]. Sometimes, there exist low contrast tumors with low ^18^F-FDG-avidity, which are easy to miss. Other times there are high physiological uptake in non-tumor regions, such as brown fat and excretory tissues/organs [17]. Therefore, appropriate segmentation of all the tumors in various patients and cancer types, while excluding normal tissues, remains a significant challenge during the automated process. To the best of our knowledge, extending such approaches to the field of tumor segmentation from whole-body PET/CT or PET/MRI has previously not been investigated. This is probably because tumors are highly diversified, with large differences in shape, size, location and SUV, whereof some lesions can exhibit similar SUV as non-pathological tissues. These characteristics make the creation of a generalizable segmentation prior difficult.

Many supervised convolutional neural network (CNN) based segmentation methods, including the widely used UNET [30] proposed by Ronneberger et al. have been developed in the past decade for biomedical image segmentation. Their encoder-decoder based architecture with skip connections forms the basis of many state-of-the-art segmentation networks that exist today. The skip connections are useful for preserving the full spatial resolution of the input. Zhou et al. later modified the plain skip connections present in the UNET with so-called nested and dense skip connections to reduce the semantic gap between the encoder and decoder. The modified network, named UNET++ [31,32], was shown to achieve better results than the traditional UNET. Most recently, Isensee et al. developed the nn-UNET [33] architecture, which is self-adapting and automatically configures the entire segmentation framework, including the topological parameters of the network, task and data specific designs (such as image size and voxel spacing), without manual intervention. Moreover, nn-UNET is shown to automatically configure itself to any biomedical image segmentation task including pre-processing, network architecture, training and post-processing. Few years back, generative models such as generative adversarial network (GAN) [34] and variational auto-encoder (VAE) [35], have gained a lot of popularity in the field of image segmentation since they try to learn the underlying latent distribution and synthesize artificial but realistically appearing images, based on the learned distribution. Conditional GANs have been shown to produce fine image segmentation results for various tasks [36]. Pix2pix [37] is one such network, used extensively for medical image segmentation as it tries to learn the underlying hidden distribution in order to transform the image from one domain to another [38].

In this paper, we propose a three-stage approach for automated tumor segmentation from for e.g., whole-body PET/CT (or PET/MRI). Stage (1): A 2D segmentation network (such as UNET++) is employed to segment lesions present in the multi-directional 2D MIPs generated from the SUV volume across different angles (between Θ = 0° and Θ = 180°) with respect to the axial axis. Each 2D projection contains important information about the full 3D image. This step is inspired by radiological review of PET images in practical scenario. Stage (2): All the 2D segmentation masks, predicted per patient by the network in the previous step, are resampled and combined using the backprojection algorithm [[39], [40], [41]] into a 3D volume in order to generate TPDMs (segmentation prior), that has the same size and spacing as the original PET/CT (or PET/MRI) images. Stage (3): The TPDM is combined with the original PET/CT (or PET/MRI) and given as an additional input channel to the state-of-the-art 3D segmentation network (such as 3D UNET) [42] to potentially enhance the overall segmentation performance and detect additional independent lesions. Finally, we validated our proposed method on three cohorts with different cancer forms and different imaging criterion, such as imaging modalities, acquisition parameters, and reference segmentation approaches, to show that our method is robust, generalizable and expandable to different tumor segmentation tasks.

## 2 Data overview

Three independent cohorts named autoPET [1,43], CAR-T [44], and cHL, based on different cancer forms and imaging modalities, were used. Ethical approval for retrospective data analysis was obtained from the Regional Ethics Review Board in Uppsala for CAR-T and the Swedish Ethical Review Authority for autoPET and cHL. A summary of the three cohorts is presented in Table 1.

**Table 1:** Description of the cohorts. Average number of lesions, patient-wise average MTV (Metabolic tumor volume), and patient-wise average SUVmax (maximum standardize uptake value) for tumor regions are shown in the respective columns.

Cohort	Modality	Patients	Scans	Follow-ups	Lesions (μ ± σ)	MTV (ml) (μ ± σ)	SUVmax (μ ± σ)
autoPET	PET/CT	900	1014	Few	14 ± 4	220 ± 343	11.06 ± 7.52
CAR-T	PET/MRI	16	48	All	9 ± 2	244 ± 468	13.81 ± 11.94
cHL	PET/CT	129	129	None	7 ± 2	243 ± 257	8.88 ± 4.84

^μ^ Mean of the measurement.

^σ^ Standard deviation of the measurement.

### 2.1 autoPET

This cohort was part of the autoPET 2022 challenge [1,43,45]. The cohort consists of high quality whole-body ^18^F-FDG PET/CT scans from patients with histologically proven lymphoma (144 scans), lung cancer (167 scans), and malignant melanoma (188 scans), as well as a negative control group (513 scans), acquired at two large medical centers in Germany (University Hospital Tübingen and University Hospital of LMU). A total of 1014 examinations from 900 patients, including the follow-up scans for a subgroup, are part of the cohort. The voxel size for all the scans in this cohort are (2.04 × 2.04 × 3.00) mm^3^. All the PET/CT scans and their manual annotations are provided as 3D volumes consisting of stacks of axial slices, usually ranging from the head to the mid-thigh level, and in some cases covering the entire body as per clinical relevance. The entire dataset was manually annotated by two expert radiologists, with ten and five years of experience in hybrid imaging and machine learning. The dataset is publicly available in TCIA (The Cancer Imaging Archive) [46].

### 2.2 CAR-T

This cohort was initially obtained to study relapsed/refractory large B-cell lymphoma patients undergoing CAR T-cell immunotherapy between 2017 and 2020, at Uppsala University Hospital, Sweden [44]. The cohort consists of pre- and post-therapy high quality whole-body ^18^F-FDG PET/MRI scans from 16 patients. This cohort constitutes 48 scans, including several follow-up scans for each patient, out of which 41 scans show active lesions and the remaining 7 scans do not show any ^18^F-FDG avid lesions. The voxel size for all the scans in this cohort are (3.13 × 3.13 × 2.78) mm^3^. All the PET/MRI scans and their manual annotations were provided as 3D volumes, ranging from head to mid-thigh. The scans were manually annotated by a radiologist with three years of experience with the annotations subsequently approved by an experienced radiologist. This dataset is not publicly available.

### 2.3 cHL

This cohort consists of ^18^F-FDG PET/CT scans from 129 patients, diagnosed with classical Hodgkin's lymphoma, at Uppsala University hospital, Sweden. A total of 129 whole-body PET/CT scans are part of this cohort, with all patients being imaged at baseline before treatment. All the PET/CT scans and their manual annotations are provided as 3D volumes, usually ranging from the neck to the mid-thigh level and in some cases also extending to the entire body as per clinical relevance. The voxel size lies in the range between (1.95 × 1.95 × 2.79) to (5.47 × 5.47 × 3.27) mm^3^. All scans were analyzed using a semi-automated contouring program called ACCURATE [52], then checked and approved manually by two nuclear medicine physicians with four and 12 years of experience. This dataset is not publicly available.

## 3 Methodology

**Fig. 1:**
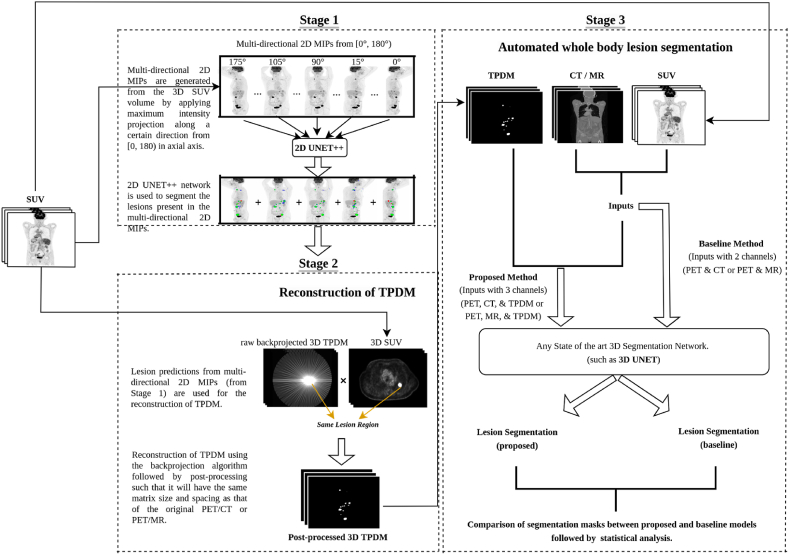
Overview of the proposed framework for automated tumor segmentation from whole-body PET/CT (positron emission tomography combined with computed tomography) or PET/MRI (positron emission tomography combined with magnetic resonance imaging). **Stage 1.** Multi-directional 2D MIP (Maximum intensity projection) based lesion segmentation from [0°, 180°) using 2D UNET++ network; **Stage 2.** All the 2D segmentation mask (predictions from stage 1) are resampled and combined into a 3D volume using the backprojection algorithm. Thereafter, voxel-wise multiplication with the original SUV (Standardized uptake value) volume is conducted to remove noise and generate the 3D TPDM (Tumor probability distribution map) of the same matrix size as that of the original PET/CT or PET/MRI; **Stage 3.** TPDM is given as an additional input channel along with PET/CT or PET/MRI to any state-of-the-art segmentation network (such as 3D UNET) for automated tumor segmentation.

The overall workflow for automated tumor segmentation from whole-body PET/CT or PET/MRI scans is shown in Fig. 1 and consists of three stages: (1) segmenting lesions in 2D MIPs, (2) reconstruction of TPDMs, and (3) tumor segmentation in 3D. All the images were pre-processed according to section 3.1. Five-fold cross validation was performed independently on each cohort for stages 1 and 3. On average each of the validation fold contained 100 (autoPET), 9 (CAR-T), and 26 (cHL) scans. The same fold-wise split was used for all the experiments.

### 3.1 Image pre-processing

In an initial step, CT scans were resampled (CTres) to their corresponding PET imaging resolution (In case of CAR-T cohort, MRI (water-fraction) scans were resampled (MRres) to their corresponding PET resolution) in order to have the same spacing. PET data was standardized by converting it into SUV (normalized by body weight). At every stage, foreground cropping was applied to remove excess background regions. Finally, voxel intensities of CTres and SUV were clipped between [−100, 250] and [0, 15] (for MRres, they were clipped between [0, 1000]) respectively, followed by intensity normalization to [0, 1]. Zero-padding and unpadding were applied according to experiment.

### 3.2 Stage 1 – multi-directional 2D MIP-based lesion segmentation

**Fig. 2:**
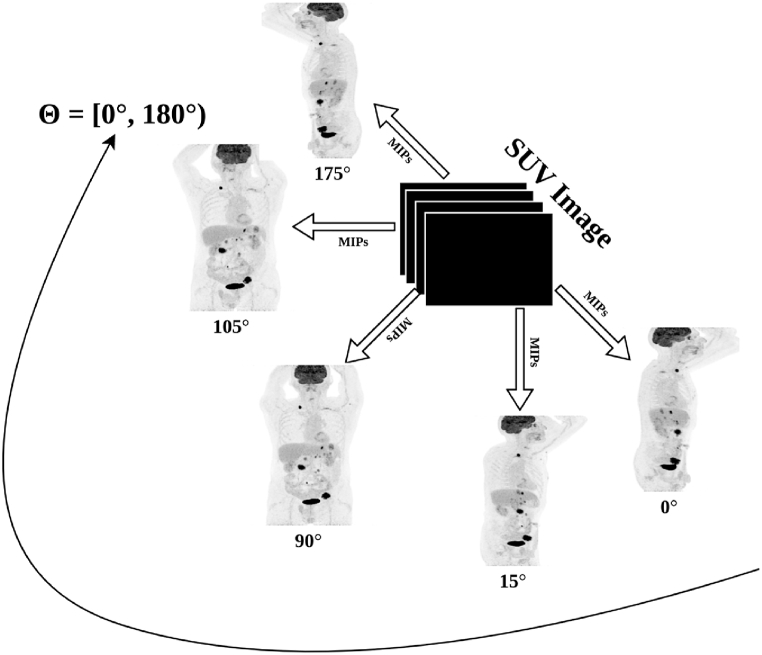
Generation of predetermined multi-directional 2D MIPs (Maximum intensity projections) between [0°, 180°) from the SUV (Standardized uptake value) image.

In the first stage, MIPs were generated from the original SUV volume, across a particular direction along the axial axis in between [0°, 180°) with an interval of 5° between each of the consecutive projections (see Fig. 2) [28]. The main reason behind using projections from [0°, 180°) and not [0°, 360°) was because the remaining projections between [180°, 360°) are going to be the mirror images of those between [0°, 180°) [40]. In total, 36 different predetermined projections were generated per patient. These projections are referred to as multi-directional 2D MIPs. Finally, the corresponding multi-directional 2D projections of the ground truth labels were generated in a similar manner, resulting in 36 ground truth projection masks corresponding to each of the multi-directional 2D MIPs per patient. These ground truth images are 2D binary masks, with the foreground pixels corresponding to the pathologies present in the multi-directional 2D MIPs and the background corresponding to the pathology-free regions.

**Fig. 3:**
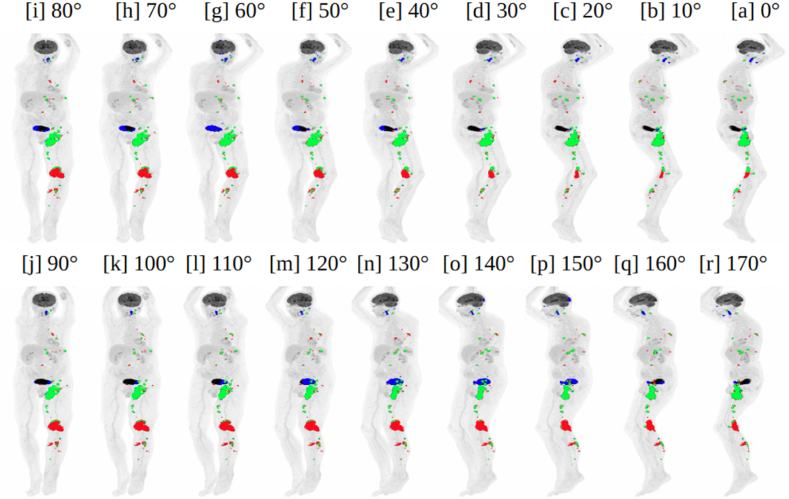
Visualization of lesion segmentation from the multi-directional 2D MIPs (Maximum intensity projections) of a patient, by the 2D UNET++ network. Figures shown in [a], [b], [c], [d], [e], [f], [g], [h], [i], [j], [k], [l], [m], [n], [o], [p], [q], [r] are examples of multi-directional 2D MIPs from a single patient. TP (True positives) voxels are shown in green, FN (False negatives) voxels in red, and FP (False positives) voxels in blue. (For interpretation of the references to colour in this figure legend, the reader is referred to the Web version of this article.)

A 2D segmentation network was then trained, using the multi-directional 2D MIPs as input, and optimized to predict the lesion segmentation within each projection image. All multi-directional 2D MIPs for a patient are treated as independent inputs to the network. The network learns to segment the pathologies within each predetermined projection, irrespective of the angle of projection and the individual patient it comes from. Fig. 3 [a] - [r] shows the robustness of the model in segmenting the lesions of different size, shape, and intensity, from the multi-directional 2D MIPs. There were a very few outlier cases where the 2D segmentation network failed to segment the lesions completely from the series of 2D MIPs. Most of them corresponded to lesions with very low SUV, often referred to as metabolically inactive lesions (see Fig. S1 in Supplementary Materials).

Three different 2D segmentation networks, UNET with residual connections [47], UNET with attention mechanism [48], and UNET++ [31,32] were first evaluated on the autoPET cohort alone using five-fold cross validation to test the efficacy of each network for the multi-directional 2D MIP-based lesion segmentation task. Subsequently, the network architecture that demonstrated the highest performance (on autoPET) was selected to conduct five-fold cross validation independently on the remaining cohorts (CAR-T and cHL). There were 18,537 (autoPET), 1517 (CAR-T), and 4773 (cHL) projection images coming from 501 (autoPET), 41 (CAR-T), and 129 (cHL) scans. All three networks were trained using the entire 2D image as input with a batch size of 1, because the 2D images are of different sizes. The overall training process was optimized by using the Dice loss function (excluding the background) [49] and Adam optimizer with a learning rate of 1e^−4^, weight decay of 1e^−5^, and a dropout factor of 0.20. The networks were validated using the following metrics: (1) Foreground Dice coefficient; (2) False negative area (FNA); (3) False positive area (FPA). Foreground Dice coefficient is defined as the total overlap between the ground truth label and segmentation prediction. FNA is defined as the average area of the connected components in ground truth that do not overlap with the estimated predictions (i.e., false negative lesions) and FPA is defined as the average area of the connected components in false positives that do not overlap with the true lesions in the ground truth (i.e., false positive lesions).

### 3.3 Stage 2 – reconstruction of TPDMs

**Fig. 4:**
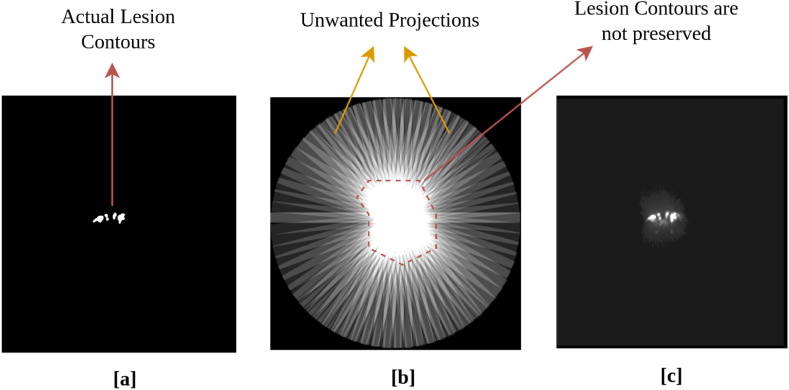
Visualization of lesions present in an axial slice. **[a]** Ground truth segmentation; **[b]** Reconstruction of TPDM (Tumor probability distribution maps) using backprojection only; **[c]** Reconstruction of TPDM using backprojection followed by post-processing.

After segmenting the lesions from the multi-directional 2D MIPs for a patient (see section 3.2), the 2D segmentation masks were combined using the backprojection algorithm [[39], [40], [41]], to reconstruct the TPDM. The TPDM is a 3D volume that has the same matrix size as the original PET/CT (PET/MRI in case of the CAR-T cohort), and contains prior information about lesion characteristics (e.g., size, shape, location, texture). During reconstruction of the TPDM, all the 2D segmentation masks (predicted by the 2D UNET++) for a patient are combined. This is accomplished by tracing all the foreground pixels (lesion predictions) from 36 different predetermined projections back to the same orientation (as that of the multi-directional 2D MIPs) and the intensity values of those projection images are copied along the same direction that was used to generate the MIP, in order to obtain 36 different 3D volumes from each of the corresponding 2D segmentation masks. Resampling, zero-padding, and un-padding is applied to all the 3D volumes in order to bring them to the same matrix size and spacing. Then they are combined into a single 3D backprojected volume (TPDM), by applying voxel-wise addition so that overlapping regions will be enhanced as compared to the non-overlapping regions. During the reconstruction of the TPDM, it becomes very important to ensure that a maximum number (ideally all) of individual lesions present are highlighted, by having some kind of prior information in the corresponding location. This simply means that all the lesions should be visible in the TPDM with adequate accuracy. One of the drawbacks of using the backprojection algorithm, to reconstruct the TPDM is that the lesion contours will not be preserved (see Fig. 4. [b]) due to the usage of binary masks during the generation of multi-directional 2D MIPs. In order to address this issue, a post-processing step was applied to the raw backprojected TPDMs, as discussed in the next section 3.3.1.

#### 3.3.1 Post-processing

**Fig. 5:**
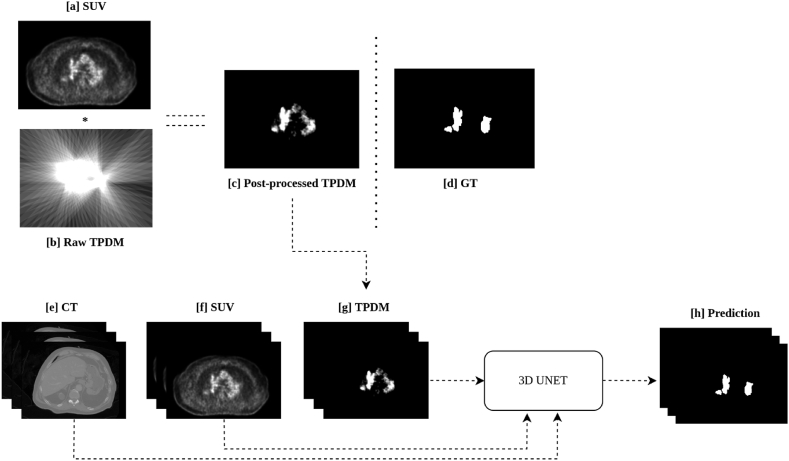
Schematic illustration of the TPDM (Tumor probability distribution map) based tumor segmentation framework. **[a]** SUV (Standardized uptake value) images and **[b]** Raw TPDM images (backprojection only) are used to create [c] the Post-processed TPDM images through backprojection followed by post-processing. [d] The Ground truth label is shown for comparison. **[e]** The 3D CT (Computed tomography) volume**, [f]** the 3D SUV volume and **[g]** the 3D TPDM volume are used as input for the prediction of **[h]** the 3D segmentation mask.

When comparing the raw backprojected TPDM volume (Fig. 4. [b]) with the corresponding ground truth segmentation (Fig. 4. [a]), the reconstructed image shows severe streaking artifacts (see Fig. 4. [b]) from the applied backprojection and very blurred segmentation boundaries (overall lesion contours are not preserved). A post-processing step, in which the raw backprojected TPDM volume is multiplied with the corresponding 3D SUV volume in a voxel-wise manner, is incorporated to fix this issue. The idea is to eliminate or minimize the unwanted artifacts, obtain relatively sharp edges at the lesion boundaries, and to improve the lesion-wise homogeneity. As the SUV volume shows higher voxel intensities in lesions compared to normal tissues, the desired characteristics of the TPDM can largely be achieved by multiplying the raw TPDM with the SUV volume (see Fig. 4. [c]). Before multiplying the SUV volume with the corresponding raw TPDM, the intensity values of the SUV volume are clipped between [0, 5] in order to give similar weighting to the majority of the lesion voxels, irrespective of lesion type and location (as lesion uptake values are usually higher than 5). Finally, the intensities of the post-processed TPDM are normalized between [0, 1] followed by removal of the bottom 5th percentile of the intensity values, mostly corresponding to noise. The final 3D volume is referred to as post-processed TPDM (see Fig. 4 [c] and Fig. 5 [c]). Despite the relatively blurry and noisy TPDM, the quality is adequate to be used as a segmentation prior.

The quality of the post-processed TPDM is evaluated using the following metrics: (1) True positive (TP); (2) False negative (FN); (3) False positive (FP). TP is defined as the average number of independent true positive lesions highlighted (visible) in the TPDM per patient. FN is defined as the average number of independent false negative lesions that are completely missed (not highlighted) in the TPDM per patient. FP is defined as the average number of independent false positive lesions per patient that do not overlap with any ground truth lesions in the TPDM. It should be noted that these metrics are lesion-based and not voxel-based.

#### 3.3.2 Impact of varying number of multi-directional 2D MIPs on the quality of TPDM

The relation between the number of projections (multi-directional 2D MIPs) and Hausdorff distance (HD95) between post-processed TPDM and corresponding ground truth label was investigated. This was done to study the change in overall quality of the TPDMs as a function of number of predetermined projections used during the reconstruction of the TPDMs, and how similar it is to the ground truth mask. For this multiple 2D UNET++ models were trained as described in section 3.2 with the number of projections equal to 1, 2, 4, 9, 18, 36 in six separate experiments for all three cohorts. The TPDMs were reconstructed according to section 3.3 using the above-mentioned number of predetermined projections. Finally, the HD95 between the TPDMs and the corresponding ground truth labels was compared in all six experiments. All the projections (in the above six experiments) were uniformly distributed between (0°, 180°).

### 3.4 Stage 3 – automated 3D tumor segmentation

In the third stage, we have used the post-processed TPDM as an additional input channel to the segmentation network for whole-body 3D tumor segmentation. A state-of-the-art 3D UNET [42] architecture with three input channels is employed, in which the first two channels correspond to the original CT and SUV (MRI and SUV in case of the CAR-T cohort) and the third channel is the post-processed TPDM as illustrated in Fig. 5.

Two state-of-the-art 3D UNETs with the same network architecture, except for the number of input channels were evaluated, first by giving two input channels (CT/MRI and SUV) and second with three input channels (CT/MRI, SUV, and TPDM), in order to study the efficacy of the TPDM. The network with two input channels is referred to as 3D UNET (baseline) and the one with three input channels is referred to as 3D UNET (proposed). The 3D UNETs (baseline and proposed) were validated separately on the three independent cohorts (autoPET, CAR-T, cHL). Five-fold cross validation was performed independently on each cohort without any data sharing between the cohorts, in order to separately validate the performance of the baseline vs proposed methods. For autoPET and cHL, the inputs to the network were SUV and CT, and for CAR-T, the inputs were SUV and MRI. Both networks (baseline and proposed) were trained using 3D patches of size (160, 160, 160), extracted according to the sliding window approach with an overlap of 0.25 between each of the consecutive patches. The overall training process was optimized by using Dice loss function (excluding the background) [49] with Adam optimizer and a learning rate of 1e^−4^, weight decay of 1e^−5^, and a dropout factor of 0.20.

### 3.5 Evaluation of predicted segmentations

The following evaluation metrics were used during five-fold cross validation [50], to compare the performance of all the segmentation algorithms between the predicted segmentation mask with the ground truth label [1]: Foreground Dice coefficient [2]; Pearson's correlation coefficient (PCC) [3]; HD95 [4]; Sensitivity (lesion-wise) [5]; Precision (lesion-wise). The Dice and HD95 scores were calculated between the ground truth segmentation and automated lesion prediction volumes. PCC was calculated from the patient-wise MTV of ground truth and prediction. Sensitivity (lesion-wise) is defined as the number of independent lesions detected (TP) divided by the total number of true independent lesions present in the body (TP + FN). Precision (lesion-wise) is defined as the number of independent lesions detected (TP) divided by the total number of independent lesions predicted (TP + FP) by the network. All lesion-wise metrics were estimated by extracting total number of independent lesions such as TP, FN, and FP from the predicted segmentation masks using cc3d module in python by finding the clusters of connected components [51] using an 18 connected neighborhood in 3D and keeping all connected components with a volume >0.5 ml.

### 3.6 Statistics

Comparison in metrics between the proposed and baseline methods were conducted using Wilcoxon signed rank test with p value < 0.05 implying statistical significance.

## 4 Results

### 4.1 Stage 1 – multi-directional 2D MIP-based lesion segmentation

Results from five-fold cross validation on the three independent cohorts are presented in Table 2. For the autoPET cohort, the highest performance was obtained with the UNET++ model. Hence, it was used as the preferred network for lesion segmentation from multi-directional 2D MIPs in all three cohorts. Fig. 3 [a] - [r] shows example images of the segmentation of different lesions present in the whole-body. In general, it was observed that the UNET++ model was capable of segmenting independent lesions from at least one or more projections (see Fig. 3 [a] - [r]). However, there were certain cases where the model failed to segment lesions in all the multi-directional 2D projections, such as for lesions with relatively low SUV (see Fig. S1 in Supplementary Materials).

**Table 2:** Performance metrics for multi-directional 2D MIP (Maximum intensity projection) based lesion segmentation in all the cohorts.

Cohort	Architecture	Metrics (μ ± σ)		
		Dice	FNA (cm^2^)	FPA (cm^2^)
	UNET (ResNets)	0.6387 ± 0.2842	19.4 ± 50.1	15.4 ± 27.1
autoPET	UNET (Attention)	0.6153 ± 0.2916	20.7 ± 45.1	29.8 ± 45.1
	**UNET++**	**0.6504 ± 0.2285**	**15.1 ± 39.1**	**12.2 ± 30.1**
CAR-T	**UNET++**	**0.4211 ± 0.3297**	**22.2 ± 47.6**	**45.6 ± 60.9**
cHL	**UNET++**	**0.7448 ± 0.2177**	**10.3 ± 17.1**	**10.8 ± 21.6**

^μ^ Mean of the measurement.

^σ^ Standard deviation of the measurement.

### 4.2 Stage 2 – reconstruction of TPDMs

**Fig. 6:**
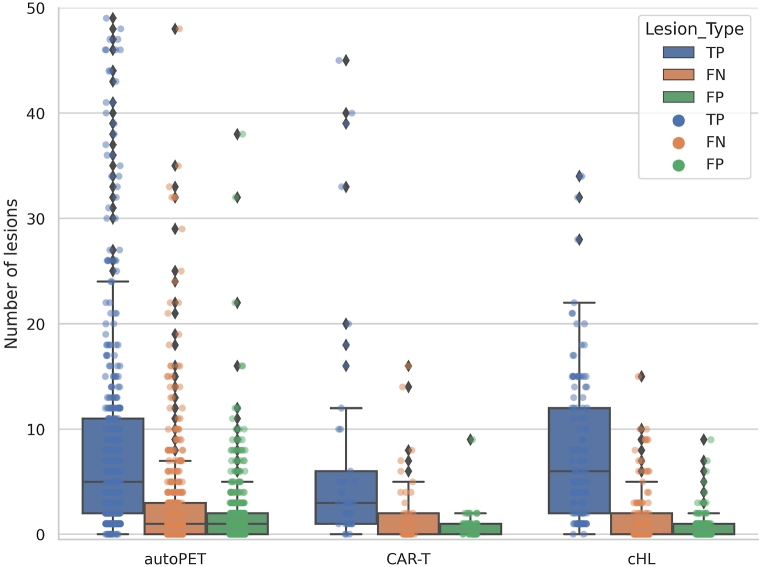
Patient-wise box plot with jitter point illustration of the number of independent true positive (TP), false negative (FN), and false positive (FP) lesions that are highlighted in the reconstructed post-processed TPDM (Tumor probability distribution map) in all three cohorts (autoPET, CAR-T, cHL). In the above plot, each dot represents a particular patient. Jitter points with the number of lesions >50 (outliers) are not shown in this plot. Number of outliers are: autoPET = 30 (TP), 2 (FN), 0 (FP); CAR-T = 0 (TP, FN, FP); cHL = 2 (TP), 0 (FN, FP).

Fig. 6 shows the grouped box plot with jitter point illustration of the number of independent TP, FN, and FP lesions highlighted in the reconstructed post-processed TPDM. The number of TPs outnumbered the number of corresponding FN and FP lesions, in all three cohorts. Multiplication of the 3D SUV with the corresponding raw TPDM volume in the post-processing step largely helped suppressing the number of FP lesions. Overall, the average number of independent FN lesions were relatively low (<3 in autoPET; <2 in CAR-T; <1.5 in cHL) (see Fig. 6), which resulted in a better reconstruction of TPDM. During the reconstruction of the TPDM, it was also observed that TPDM contains additional valuable information in order to be used as a segmentation prior. However, there are few cases where the TPDM is empty because the 2D UNET++ network in section 3.1 failed to segment any lesion. Hence, there will not be any overlapping regions after reconstruction (no prior information). The number of cases with empty TPDM are 3 (autoPET), 0 (CAR-T), and 1 (cHL).

**Fig. 7:**
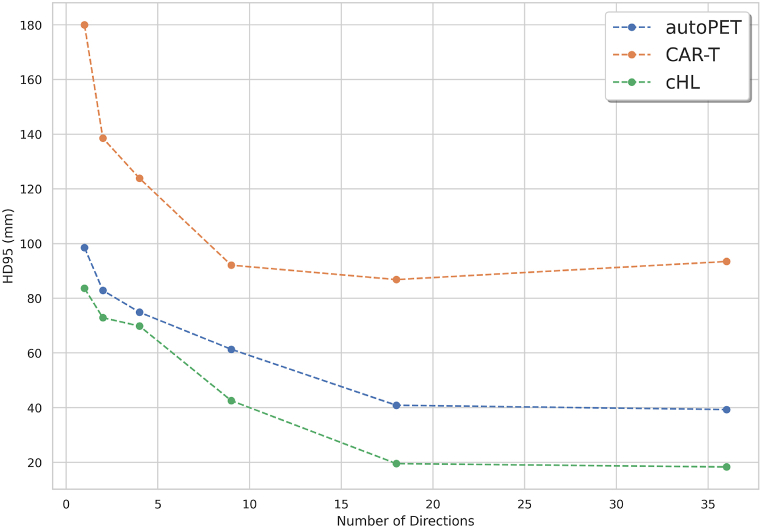
Illustration of the number of projections (multi-directional 2D MIPs) used to reconstruct the TPDM (Tumor probability distribution map) vs HD95 (Hausdorff distance) between the ground truth label and the corresponding TPDM in all three cohorts (autoPET, CAR-T, cHL).

It is evident from Fig. 7 that the best quality TPDMs can be reconstructed with the number of projections equal to 18. As anticipated, HD95 initially decreases sharply with increased number of projection angles and eventually saturates when the quality of TPDM no longer can be enhanced.

### 4.3 Stage 3 – automated 3D tumor segmentation

The results of the baseline vs proposed methods for all the employed cohorts are presented in Table 3. In general, the median Dice coefficient is higher than the mean Dice in all the cohorts, which signifies that the patient-wise Dice in most cases is higher than the average. There are a few cases with zero or very low Dice coefficients, affecting the overall mean Dice.

**Table 3:** Comparison of performance metrics between 3D UNET (baseline) vs 3D UNET (proposed) for automated tumor segmentation in all the cohorts.

Cohort	Network Architecture	Dice (μ ± σ)		PCC	HD95 (mm) (μ ± σ)	Sensitivity (Lesion-wise)	Precision (Lesion-wise)
		mean	median				
autoPET	3D UNET (baseline)	0.6750 ± 0.2507	0.7628 ± 0.2507	0.8859	43.52 **±** 65.50	0.7253	0.7686
	**3D UNET (proposed)**	**0.7001 ± 0.2210**	**0.7788** ± **0.2210**	**0.9037**	**38.92 ± 59.73**	**0.7833**	**0.8437**
CAR-T	3D UNET (baseline)	0.3573 ± 0.3049	0.3618 ± 0.3049	0.9001	79.31 **±** 66.42	0.5579	0.5178
	**3D UNET (proposed)**	**0.5012 ± 0.3198**	**0.5963 ± 0.3198**	**0.9470**	**70.02 ± 68.67**	**0.6538**	**0.6939**
cHL	3D UNET (baseline)	0.7218 ± 0.2092	0.7853 ± 0.2092	0.8494	18.54 **±** 31.17	0.8045	**0.8082**
	**3D UNET (proposed)**	**0.7746 ± 0.2071**	**0.8518 ± 0.2071**	**0.9019**	**16.09 ± 28.90**	**0.8821**	0.7972

^μ^ Mean of the measurement.

^σ^ Standard deviation of the measurement.

**Fig. 8:**
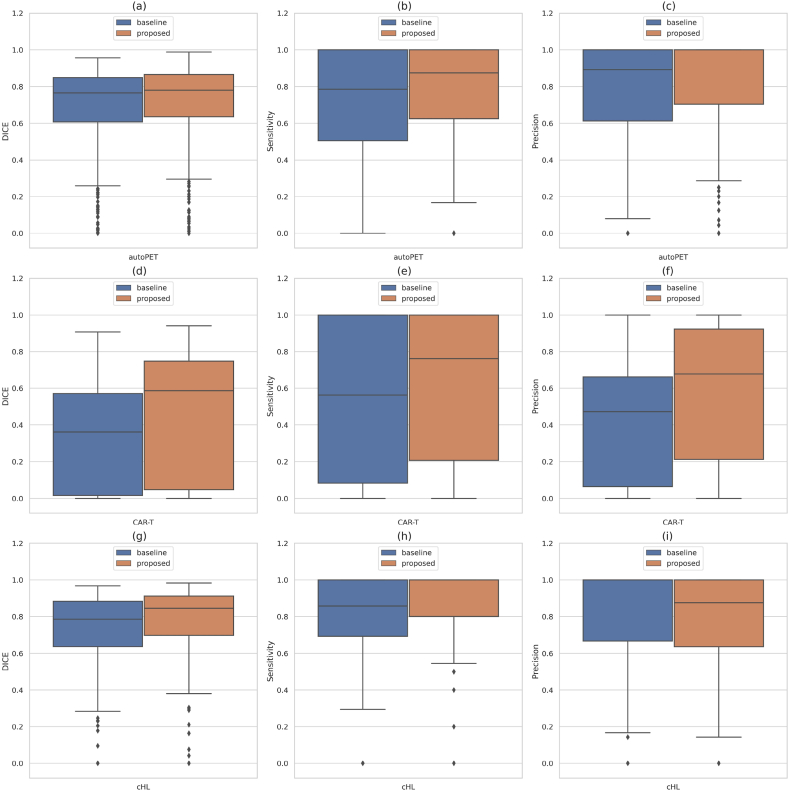
Box-plot visualization of performance metrics such as Dice, sensitivity (lesion-wise), and precision (lesion-wise) for baseline and proposed models in all the cohorts. [a], [b], [c] corresponds to the autoPET; [d], [e], [f] corresponds to the CAR-T; and [g], [h], [i] corresponds to the cHL cohort.

Comparisons between the baseline and proposed models for all three cohorts are shown in Fig. 8 [a] - [i], with each subplot illustrating the box plot distribution of the performance metrics such as Dice coefficient, sensitivity (lesion-wise), and precision (lesion-wise) for each cohort. It is evident from Table 3 and Fig. 8 [a] - [i] that the proposed model outperforms the baseline model in terms of all the criteria.

Finally, in order to test the statistical significance between the baseline and proposed models, Wilcoxon signed rank test was used as a non-parametric method. Significant improvement occurs for all cancer types as illustrated in Table 4. A similar trend was observed in all the cohorts, especially for CAR-T and cHL where the proposed model outperformed the baseline model with relatively high margins (see Table 3).

**Table 4:** Statistical comparison of Dice, sensitivity (lesion-wise), precision (lesion-wise) between the baseline and proposed models using the Wilcoxon signed rank test.

Cohort	p-value		
	Dice	Sensitivity	Precision
autoPET	0.0001	<0.0001	<0.0001
CAR-T	0.0096	0.0800	0.0023
cHL	<0.0001	<0.0001	0.1452

**Fig. 9:**
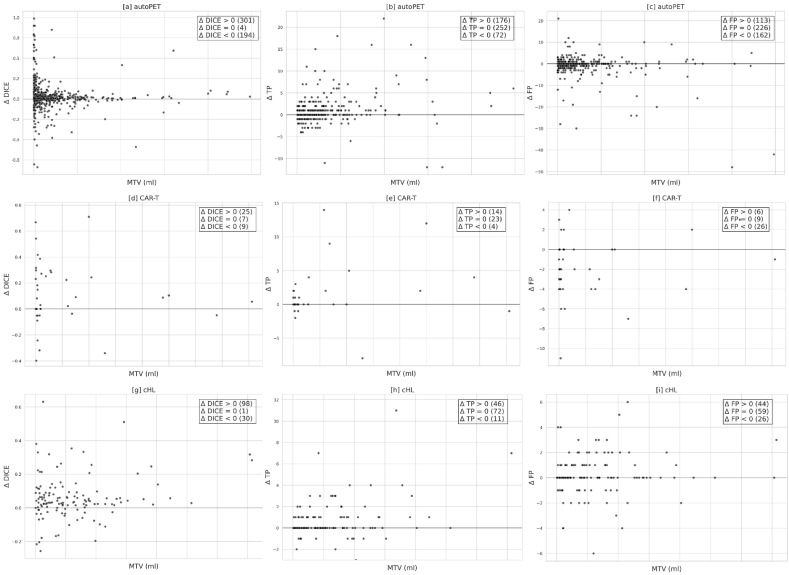
Scatter plot illustration for patient-wise MTV (metabolic tumor volume) vs Δ Dice, Δ TP, Δ FP, for all the three cohorts, where Δ metrics = metric (proposed) – metric (baseline). [a], [d], [g] corresponds to MTV vs Δ Dice. [b], [e], [h] correspond to MTV vs Δ TP. [c], [f], [i] correspond to MTV vs Δ FP for all the three cohorts. Here, TP and FP correspond to the total number of true positive and false positive lesions. In the above plots, each dot represents a particular patient.

Fig. 9 [a] - [i] correspond to the patient-wise plots between the MTV and Δ Dice, Δ TP, and Δ FP, in all three cohorts. It is evident from the plots that Δ Dice and Δ TP are increasing, whereas Δ FP is decreasing for most cases after employing the proposed method.

**Fig. 10:**
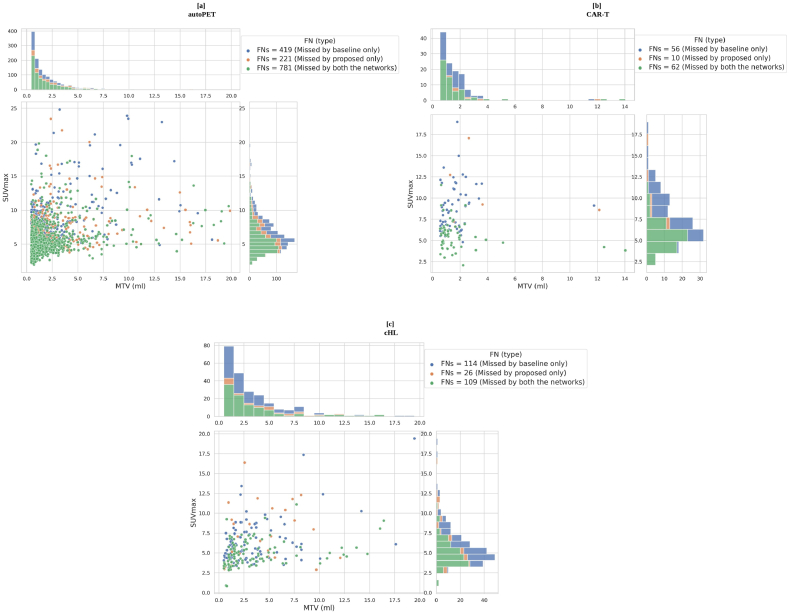
Illustration of scatter plots with marginal histogram for lesion-wise MTV (Metabolic tumor volume) vs SUVmax (Maximum standardized uptake value) for independent FN (False negative) lesions present in all three cohorts for both methods. [a] corresponds to autoPET, [b] corresponds to CAR-T, and [c] corresponds to cHL cohort. In the above plots, each dot (shown in blue, green, and orange) represents a particular lesion. Blue dots correspond to FN lesions by the baseline network only, that are picked up by the proposed network. Green dots correspond to FN lesions by both the networks. Orange dots correspond to FN lesion by the proposed network only, which are picked up by the baseline network. The histograms visualize the corresponding distribution of the lesions, with respect to SUVmax and MTV. (For interpretation of the references to colour in this figure legend, the reader is referred to the Web version of this article.)

Fig. 10 [a] - [c] illustrates the lesion-wise scatter plots with marginal histogram for the MTV vs SUVmax for all the independent false negative lesions, by both models. Here, blue dots represent the lesions missed by the baseline model, which are picked up by the proposed model. Green dots are the lesions that are missed by both the models. Orange dots (outliers) are the lesions missed by the proposed model but picked up the baseline model. In general, most of the difficult to segment lesions (by the baseline model) either have small MTV or low SUV, or both.

**Fig. 11:**
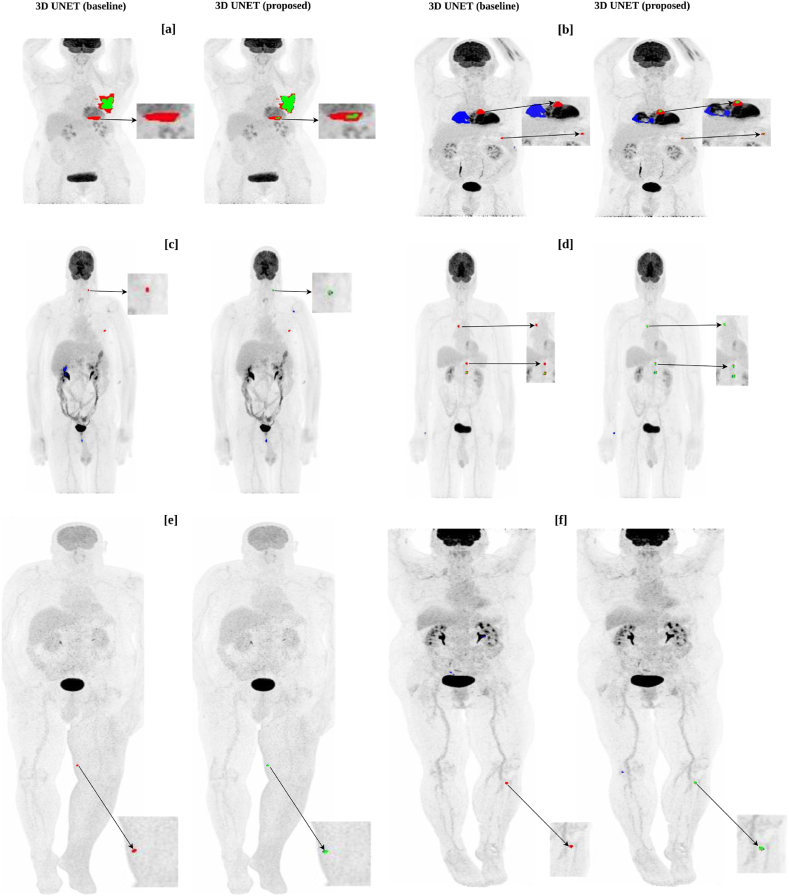
Visualization of lesion predictions that are completely missed by the 3D UNET (baseline) model but are picked up by the 3D UNET (proposed) model. Figures shown in [a], [b], [c], [d], [e], [f] are examples of lesions that are difficult to segment, from all the three cohorts, because of their extremely small size. TPs (True positives) are shown in green, FNs (false negatives) in red, FPs (false positives) in blue. (For interpretation of the references to colour in this figure legend, the reader is referred to the Web version of this article.)

Fig. 11 [a] - [f] shows the visualization of patients with lesions difficult to segment (especially extremely small ones) that are missed by the 3D UNET (baseline) model but are detected by the 3D UNET (proposed) model.

The training times for both, the baseline and the proposed models were comparable. The proposed model took little longer, primarily due to additional computational requirements, associated with the process of reconstruction of the TPDM. Specifically, for the autoPET cohort, the five-fold cross validation took 120 h for the baseline and 125 h for the proposed model, for the CAR-T cohort, it was 20 and 22.5 h, and for the cHL cohort, it was 25 and 27.5 h, respectively.

## 5 Discussion

In this study, we propose a novel approach to deep learning-based tumor segmentation of whole-body PET/CT and PET/MR images, by incorporating TPDMs as segmentation priors into the overall framework. The TPDM was given as an additional input channel along with the original PET/CT or PET/MRI, to the 3D UNET in order to enhance the overall segmentation and provide improved delineations. We believe that integrating such segmentation priors (TPDM) into the 3D UNET will guide the network towards predicting more meaningful delineations. One of the main objectives of this study was the accurate reconstruction of TPDMs, providing detailed prior information about the target lesions. We found that this approach improved the overall accuracy in identifying TP tumors while reducing FPs from non-cancerous tissues. The 3D UNET (proposed) was able to segment difficult tumors after the incorporation of TPDMs, while the structures that could have been mistaken for a lesion were correctly ignored. This method was very efficient in detecting tumors in the multi-directional 2D MIPs, resulting in a TPDM highlighting the majority of the lesions in the body. However, there were a few cases where the TPDM was empty. Segmentation accuracy for the proposed model in these cases was identical to the baseline model.

In the context of TPDM reconstruction, training a 2D segmentation network to estimate the approximate location of lesions (especially extremely small lesions) is a much simpler task compared to training a 3D segmentation network for the same purpose. This is due to the lower class-imbalance between foreground (tumor) and background (normal) voxels and smaller ambiguity at tumor boundaries. Also, the process of creating maximum intensity projections from various angles and then backprojecting them into a unified 3D volume, completely transforms the original 3D SUV volume into a separate and complementary input volume. As we are segmenting the same tumors from multi-directional 2D MIPs, the tumors which are overlooked by the network from a certain direction, could possibly be segmented from a different direction. Fig. 3 [a] - [r] shows that lesions that were difficult to segment (completely missed by the network) from a certain direction (shown in red), were possible to segment from a different direction (shown in green). The regions incorrectly segmented as lesions (shown in blue) in a few projections, were also successfully ignored when viewed in other projections. In general, the 2D segmentation network was able to segment the majority of lesions including the difficult ones (mostly small lesions), in one or more projections. Ultimately, it can be used to estimate the average location of the lesions present in the corresponding 3D volume. Although this 2D projection-based approach will not provide a final 3D segmentation mask, it creates the basis of our proposed method, from which a 3D segmentation mask can be generated.

Previous studies have shown that incorporation of domain-specific knowledge of the target lesions (such as size, shape, texture, boundary, contrast, and appearance), within the segmentation framework, can enhance the overall performance [[25], [26], [27]]. This additional information is often referred to as a segmentation prior. In a recent study, Astaraki et al. proposed to model the appearance of healthy lungs in CT images, and use it to generate a segmentation prior that contains information about the corresponding anomalies and use it for lung pathology segmentation [23,24]. In another study, Angermann et al. proposed to use maximum intensity projections (MIPs) from different orientations in order to transform the three-dimensional (3D) data to a series of two-dimensional (2D) images. This was followed by automated segmentation of blood vessels from the 2D MIPs and finally backprojecting to the 3D space, using a trainable reconstruction algorithm [28]. Pan et al. also used a similar approach in which they generated MIPs from three different directions (axial, sagittal, and coronal) followed by tumor segmentation in 2D and reconstruction of 3D segmentation masks for comprehensive tumor metastasis analysis [29]. Most recently, during the autoPET 2022 challenge, whole-body PET/CT based tumor segmentation methods achieved an overall Dice scores ranging from 60 to 72% [53].

Often, extremely small lesions were completely missed by the 3D UNET (baseline) network due to uncertainty during the overall optimization process and because of design parameters. By incorporating meaningful prior information about the target lesions, this issue can to some degree be solved (see Fig. 11 [a] - [f]). Our proposed model produces more robust segmentation results when the tumor regions are highlighted in the TPDM. On the other hand, when the tumors are not highlighted, the proposed model proceeds with the segmentation task with no useful prior information, resulting in a segmentation similar to the baseline model. During inference, we found that the number of cases with empty TPDMs were 3 (autoPET), 0 (CAR-T), and 1 (cHL). Segmentation accuracy for these cases was identical to the baseline model. Fig. S1 in the Supplementary Materials shows one such example of an outlier case with empty TPDM because the 2D segmentation network (as described in Section 3.2) failed to segment the lesion completely from the multi-directional 2D MIPs. Due to this, the TPDM did not contain any additional valuable information, resulting in zero improvement in terms of overall 3D segmentation accuracy for the patient. We validated our proposed method on three independent cohorts with different imaging criterion in terms of imaging modalities, image acquisition parameters, cancer types, etc in order to compare the efficiency of the proposed method with the baseline. It can be observed from Table 3 that integrating the TPDM into the segmentation framework, successfully improved the overall segmentation performance in all the three cohorts. In fact, the median performance of the proposed model was higher than those of the baseline in all the cohorts (see Fig. 8 [a] - [i]).

The supremacy of the proposed method over the baseline can also be verified from the plots shown in Fig. 9, Fig. 10. Fig. 9 illustrates the patient-wise scatter plots (Each dot represents a particular patient) for MTV vs change in Dice and the number of TP and FP, between the proposed and baseline methods in all three cohorts. An increase in the overall patient-wise Dice coefficient and the number of TP lesions were observed. Simultaneously, a decrease in the number of FP lesions was also noticed. Combined, this highlights that the proposed method outperformed the baseline method in a patient-wise manner. Fig. 10 corresponds to the lesion-wise scatter plots with marginal histogram (Each dot represents a particular lesion) for MTV vs SUVmax for all the independent FN lesions by both methods. Although, most of the lesions (FN) missed by the baseline method (shown as blue dots) were picked up by the proposed method (autoPET = 419; CAR-T = 56; cHL = 114), further visualized in Fig. 11 [a] - [f]. There remain few lesions (FN) missed by the proposed method (shown as orange dots) but picked up by the baseline method (autoPET = 221; CAR-T = 10; cHL = 26). They can be thought of as outliers due to study design and optimization parameters. In short, the proposed method is able to detect a greater number of independent lesions compared to the baseline method with no additional FPs, resulting in an increased lesion-wise sensitivity and precision. However, there remain some lesions (FN), missed by both networks (shown as green dots) (autoPET = 781; CAR-T = 62; cHL = 109). As stated previously, if a lesion is relatively large (typically, MTV >2 ml) with high enough SUV, it is relatively easy to segment with adequate accuracy. However, if a lesion is extremely small (typically, MTV <2 ml), it is often missed by the baseline model irrespective of the SUV. It is evident from the plots (see Fig. 10) that the proposed method can detect many of these extremely small lesions with high enough SUV (typically, SUVmax >4), previously missed by the baseline model. However, there remain some cases where both models failed to segment the target lesions, most of which correspond to lesions with very low SUV, e.g., as they are at an early stage of cancer or because the lesions are no more metabolically active over the course of therapy response. It is also important to note that patient-wise (as shown in Fig. 9) and lesion-wise (as shown in Fig. 10) metrics are not the same. E.g., lesion-wise MTV is the MTV of each lesion whereas patient-wise MTV is the MTV of each patient (which can have multiple lesions).

It is also evident from Fig. S2 in the Supplementary Materials that most of the independent FN lesions have low SUV as compared to the TP lesions. Moreover, the FP predictions often correspond to lesion-like structures with extremely high SUV, which are not actual lesions but e.g., excretory wastes, inflammation, and artifacts. One aspect of our future work will be towards trying to enhance the segmentation accuracy of such FN lesions with very low SUV and minimize the overall FP predictions. During the follow-up analysis, we found that some of the FP predictions by the proposed network are true lesions that are missed by the human annotator during the initial ground truth assessment either because of their extremely small size or due to human error. Detecting these additional lesions manually would require a time intensive and laborious re-examination of the entire scans, taking several weeks of manual work. Instead, this can be achieved by increasing the detection rate (sensitivity) of the proposed method. This also increases the rate of FPs, however, correcting these errors only requires a human annotator to review the predictions, which can be done in a few minutes (per patient) of time. Hence, having a segmentation model with high sensitivity is clinically more viable.

The Dice coefficient alone is not necessarily the best metric to use during the evaluation of tumor segmentation tasks as it does not take into account the number of independent lesions detected. In theory, a model can have a reasonably high Dice coefficient despite failing to segment many small lesions as their contribution to the overall Dice would be low. This has occurred with the 3D UNET (baseline) model (see Fig. 11 [a] - [f]), which failed to detect the presence of many small lesions, resulting in a relatively high Dice coefficient but a lower sensitivity (lesion-wise) (see Table 3). Detection and segmentation of small lesions are clinically very important, especially in follow-up investigations where a missed small new lesion represent progressive disease because of therapeutic failure. Our proposed method specifically focuses on detecting those small lesions with reasonable accuracy. To the best of our knowledge, most articles on tumor segmentation focus solely on voxel-wise Dice coefficient as the primary metric, thereby neglecting the importance of optimizing the number of true lesions detected. Therefore, we have included the lesion-wise sensitivity and precision as additional performance metrics during the evaluation (see Table 3), to make a more clinically relevant comparison between the baseline and proposed models.

A significant improvement in the overall Dice coefficient is observed in the CAR-T cohort (Δ = +0.1440, p = 0.0096), whereas in the case of autoPET, the improvement is numerically smaller although statistically significant (Δ = +0.0251, p = 0.0001). On the other hand, a moderate improvement can be seen in the cHL cohort (Δ = +0.0528, p < 0.0001). This is because the majority of lesions present in the CAR-T cohort are small compared to in the autoPET cohort, where the small lesions occupy a minor portion of the total number of independent lesions. As a result, although the proposed model detects a larger number of true lesions in the autoPET cohort (indicated by increased lesion-wise sensitivity), the increase in Dice is less prominent. In the cHL cohort, small lesions constitute a large portion of the lesions, but they are not in majority, unlike in the CAR-T cohort. It is clear from Table 3 that the increase in overall lesion-wise sensitivity is significantly higher (in autoPET Δ = +0.0580, p < 0.0001; in CAR-T Δ = +0.0959, p = 0.0799; in cHL Δ = +0.0776, p < 0.0001) for the proposed method, compared to the baseline method. This implies that the proposed model can detect more true lesions with reasonable accuracy than the baseline model. However, such Dice scores might be considered sufficient for diagnosing tumors, at least helpful as an independent second opinion or initial suggestion to the radiologist/nuclear medicine physician, but not for basing treatment planning on.

From a clinical perspective, it is likely more important to detect many lesions (especially the small and low-SUV lesions easily escaping the human eye during reviewing) without perfect segmentation of each lesion, than obtaining a mean high Dice coefficient across all lesions meanwhile missing several small lesions. One important thing to notice is that the overall lesion-wise sensitivity does not increase at the cost of precision. In fact, the overall lesion-wise precision also increases after employing the proposed method in autoPET (Δ = +0.0751, p < 0.0001) and CAR-T (Δ = +0.1761, p = 0.0023) cohorts, except cHL, where a slight drop (Δ = −0.011, p = 0.1452) of around 1% is noticed (see Table 3, Table 4). This means that the number of FP predictions decreases in most cases after employing the proposed method. One reason for this could be the way TPDMs are reconstructed from the multi-directional 2D MIPs, since the non-pathological structures that could have been mistaken for a lesion in a single 2D projection was correctly ignored during the reconstruction process. It is relatively easy to avoid segmenting the non-pathological regions with extremely high SUV from multi-directional 2D MIPs than from the 3D volume. Therefore, the non-pathological regions (that are previously picked up as lesions by the baseline model) are no longer highlighted in the TPDM, resulting in fewer FPs by the proposed model. In short, having a TPDM not only highlights the presence of additional small lesions present in the 3D scan but also helps reducing the unnecessary FP predictions by not highlighting the non-pathological regions that are previously picked up as tumor lesions by the baseline model.

The proposed method holds potential for providing valuable assistance to radiologists/nuclear medicine physicians by facilitating and streamlining the process of tumor detection and annotation. The method can provide an independent second opinion or an initial tumor suggestion, which subsequently can be verified and adjusted by the human interpretor if needed. Morever, the proposed method demonstrates the capability to detect additional small lesions, which are easily overlooked during visual assessment. Segmenting the lesions from the multi-directional 2D MIPs and then reconstructing the TPDM is a convenient and cheap way, in terms of computing power and time, of estimating the overall location of lesions present in the 3D images. In the context of deep learning, the TPDM can be considered as an attention weight map, which can be used along with the original PET/CT or PET/MRI to specify the importance of different regions within the 3D scan. Using the TPDM is an indirect way of drawing attention towards the lesion regions, in order to reduce the overall uncertainty, present in the baseline network and thus creating more meaningful delineations. This can also be verified with the results presented in Table 3 and Fig. 8 [a] - [i], where the proposed method outperforms the baseline with considerable margins in all the cohorts for all types of cancer forms.

Our proposed method was initially developed using the autoPET cohort and thereafter extended to the CAR-T and cHL cohorts in order to test the reliability and generalizability of the proposed framework. Among the three cohorts, the segmentation performance for both models (baseline and proposed) was comparatively poor (see Table 3) for CAR-T because of the relatively large number of small and low SUV lesions. This is due to the examinations mostly consisting of follow-up scans after therapy, with shrinking and disappearing tumors over time. However, the CAR-T cohort was intentionally chosen to verify the reliability of the proposed method for different lesion and imaging modalities. Moreover, tumor segmentation in PET/CT (or PET/MRI) is challenging due to the dual nature of the acquired information i.e., low contrast information in CT and low spatial resolution in PET. Nevertheless, the intention of this study was not to compete with other state-of-the-art networks but rather to verify, as proof of concept that TPDM can improve the overall segmentation performance as compared to the baseline model with similar parameters.

Finally, we also studied the relationship between the number of predetermined projections (multi-directional 2D MIPs) vs HD95 score in the TPDM and corresponding ground truth label. The main intention was to check whether the inclusion of 36 different projections was needed during the reconstruction of high quality TPDM or whether comparable results can be obtained with fewer projections. This was investigated by comparing the HD95 score between the TPDMs and the corresponding ground truth masks in order to assess how similar they are to each other. It is clear from Fig. 7 that the HD95 score initially decreased substantially with increased number of projections (used to reconstruct the TPDM) and then became saturated when the quality of the TPDM cannot be further enhanced. Similar trend was observed in all the cohorts. Since the quality of the TPDM has a direct impact on the overall 3D segmentation accuracy, these results indicate that superior performance can be achieved with only 18 projections compared to 36 (see Fig. 7). The reason for this was saturation. After a certain number of projection angles, the quality of the TPDM was no longer improved as the overall coverage with 18 projections was very similar to that of 36 projections. This ultimately results in improved 3D segmentation performance with less training (2D UNET++) and reconstruction time after integrating the high quality TPDM within the final segmentation framework.

One of the major drawbacks of the proposed method was that it failed to show considerable improvement compared to the baseline method for lesions exhibiting low SUV (SUVmax <4), irrespective of their size (see Fig. 10). This was due to the poor contrast between non-metabolically active lesions and background in the PET images. Sometimes, multiple lesions may be present close to regions with high physiological uptake, such as the brain, bladder, heart etc, which makes it very challenging for the automated segmentation model (both baseline and proposed models) to detect these lesions. This might mean that the network gets biased about these regions after seeing a lot of instances where physiologically high uptake regions usually correspond to normal tissue (see Fig. S3 in Supplementary Materials). Another limitation of the study was the small sample size of the CAR-T cohort. Despite this, statistically significant improvements in terms of Dice coefficient and lesion-wise sensitivity and precision were obtained. Lastly, the ground truth annotations were based on manual delineations performed by different operators, which resulted in relatively large variations in the lesion annotations. Modifications resulting in increased standardization and more objective ground truth segmentation might improve the performance of the segmentation methods and could be a future opportunity for improvement.

In future work, we would like to further enhance the sensitivity of the proposed model towards low SUV lesions without any major false positives. We also plan to improve the reconstruction of the TPDM to enhance overall 3D segmentation accuracy. Finally, we would also like to train a CT or MRI-only model, for segmentation of the tumors based only on CT or MR images. This will be clinically helpful as PET is not as widespread as CT and MRI. In summary, this work contributes to the advancements within the field of deep learning-based tumor segmentation with an important long-term goal of incorporating automated tumor segmentation solutions into clinical practice.

## 6 Conclusion

In this paper, we propose a novel method for enhancing deep learning based segmentation of tumor lesions by reconstructing and incorporating a TPDM as segmentation prior along with the original whole-body PET/CT or PET/MR images. We evaluated the performance on three independent cohorts, based on different cancer forms, and acquired with different imaging parameters and modalities, in order to show that the proposed method is truly effective in improving the overall segmentation accuracy compared to the baseline model and easily can be extended to other datasets. The segmentation results showed that the proposed method outperformed the baseline method in all the cohorts and for all cancer types, with many lesions difficult to segment and previously missed by the baseline network being detected by the proposed network. From this comparison, it can be concluded that integrating such a TPDM as segmentation prior can successfully improve the segmentation performance of the network.

## Declarations

### Ethics approval and consent to participate

Ethical approval for retrospective data analysis was obtained from the Regional Ethics Review Board in Uppsala for CAR-T (Reference number Dnr 2017/524 for the healthy volunteer cohort and Dnr 2017/449 for the lymphoma cohort) and the Swedish Ethical Review Authority for autoPET (Reference number Dnr 2023-02312-02) and cHL (Dnr 2011/132) cohorts. The study was conducted in accordance with relevant guidelines and regulations, including the Declaration of Helsinki.

### Consent for publication

Not applicable.

### Availability of data

The autoPET dataset is publicly available at The Cancer Imaging Archive (TCIA). CAR-T and cHL are private datasets that may be made available on reasonable request.

### Competing interests

Joel Kullberg and Håkan Ahlström reports a relationship with Antaros Medical AB that includes: employment and equity or stocks. Joel Kullberg also has patent pending to Assignee. All other authors have no competing interests.

### Funding

This study was supported by 10.13039/501100002794Swedish Cancer Society (201,303 PjF 01 H), Lions Cancer Fund Uppsala and Makarna Eriksson foundation. For cHL cohort, additional support was provided by the Steno Diabetes Center Aarhus (SDCA) which is partially funded by an unrestricted donation from the 10.13039/501100009708Novo Nordisk Foundation.

## CRediT authorship contribution statement

**Sambit Tarai:** Writing – review & editing, Writing – original draft, Visualization, Validation, Methodology, Investigation, Formal analysis, Conceptualization. **Elin Lundström:** Writing – review & editing, Visualization, Validation, Supervision, Project administration, Investigation. **Therese Sjöholm:** Writing – review & editing, Visualization, Validation, Investigation. **Hanna Jönsson:** Writing – review & editing, Visualization, Investigation. **Alexander Korenyushkin:** Writing – review & editing, Visualization. **Nouman Ahmad:** Writing – review & editing. **Mette A. Pedersen:** Writing – review & editing, Data curation. **Daniel Molin:** Writing – review & editing, Data curation. **Gunilla Enblad:** Writing – review & editing, Data curation. **Robin Strand:** Writing – review & editing, Validation, Supervision. **Håkan Ahlström:** Writing – review & editing, Supervision, Project administration, Funding acquisition, Data curation. **Joel Kullberg:** Writing – review & editing, Visualization, Validation, Supervision, Project administration, Methodology, Investigation, Funding acquisition, Conceptualization.

## Declaration of competing interest

The authors declare the following financial interests/personal relationships which may be considered as potential competing interests: Joel Kullberg reports a relationship with Antaros Medical AB that includes: employment and equity or stocks. Hakan Ahlstrom reports a relationship with Antaros Medical AB that includes: employment and equity or stocks. Joel Kullberg has patent pending to Assignee.
