# Supplementary material for: Improved automated tumor segmentation in whole-body 3D scans using multi-directional 2D projection-based priors
Source: Heliyon. 2024 Feb 15;10(4):e26414. doi: 10.1016/j.heliyon.2024.e26414 (PMC10882139; doi:10.1016/j.heliyon.2024.e26414)
Supplement: Multimedia component 1 [file mmc1.docx]

**Supplementary Material:**


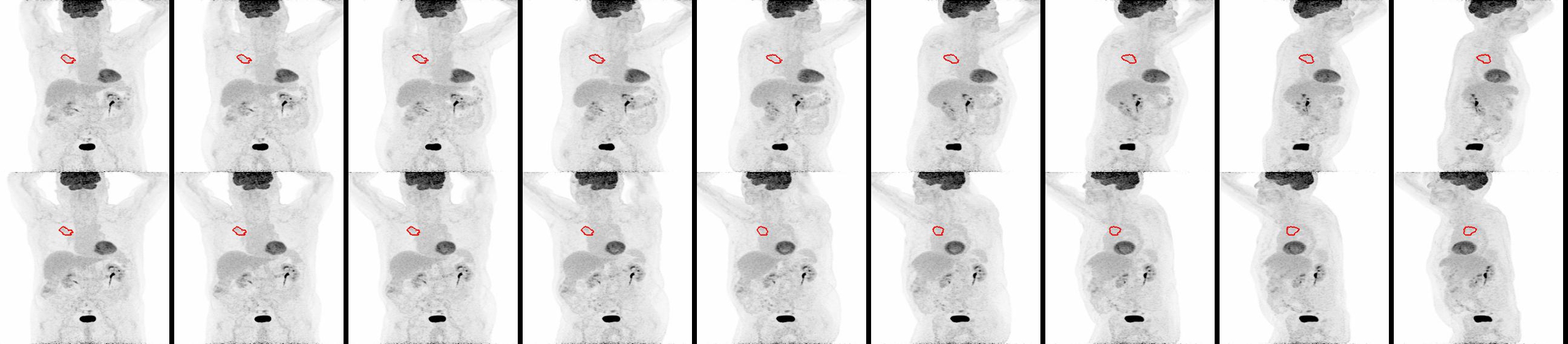


**Figure S1.** Illustration of an outlier case where the 2D UNET++ network failed to segment the lesion completely from the multi-directional 2D MIPs (Maximum intensity projections). The tumor contour is highlighted in red.


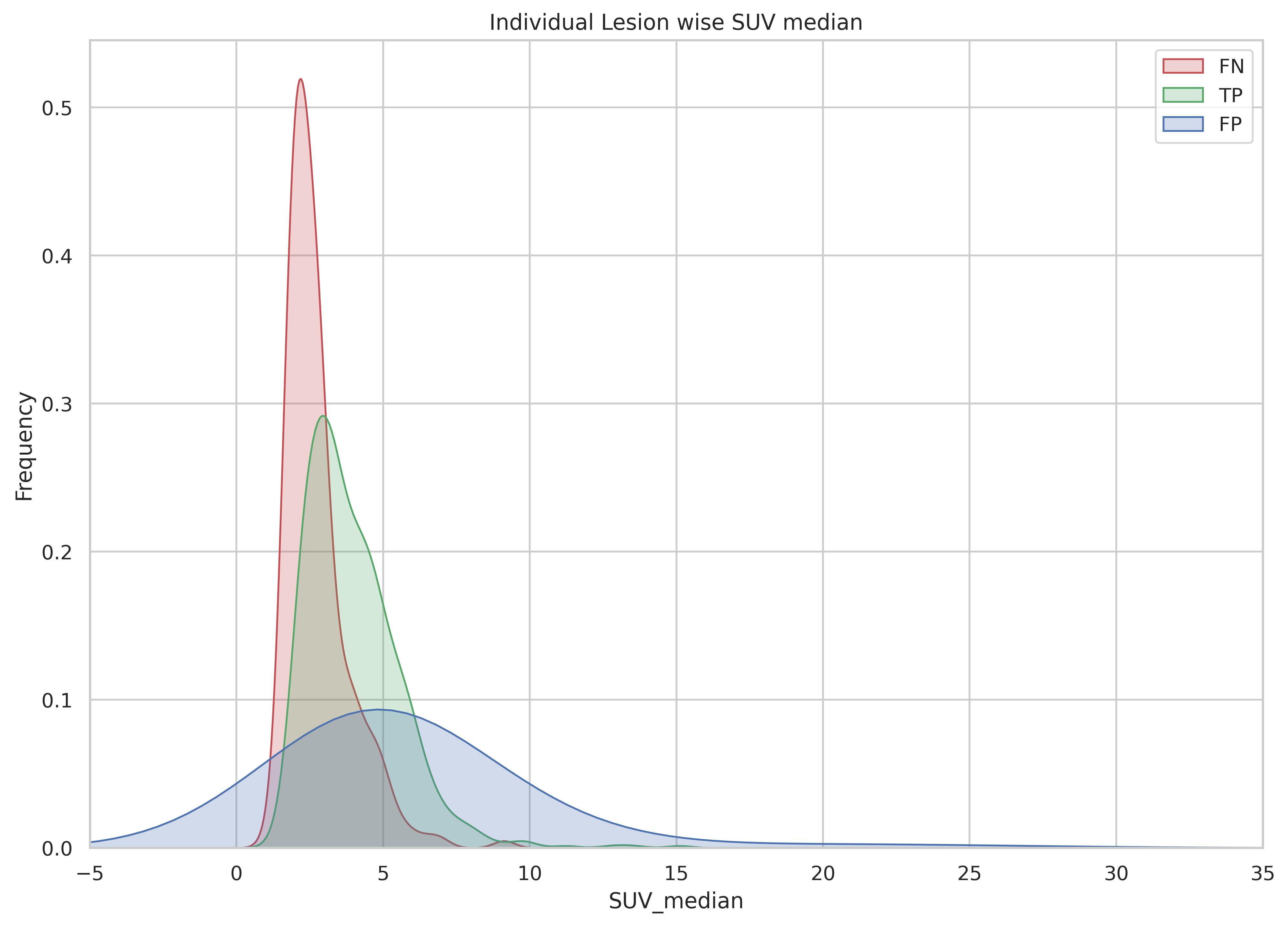


**Figure S2.** Lesion-wise distribution of independent TP (true positives), FN (false negatives), and FP (false positives) lesions over the SUV_median (Standardized uptake value) for each lesion. Distribution of TP is shown in green, FN in red, and FP in blue. The distribution was generated using the 3D UNET (proposed) method from the autoPET cohort. It is also important to note that there are no FP lesions with SUV_median less than 0, the distribution looks continuous just for visualization purpose.


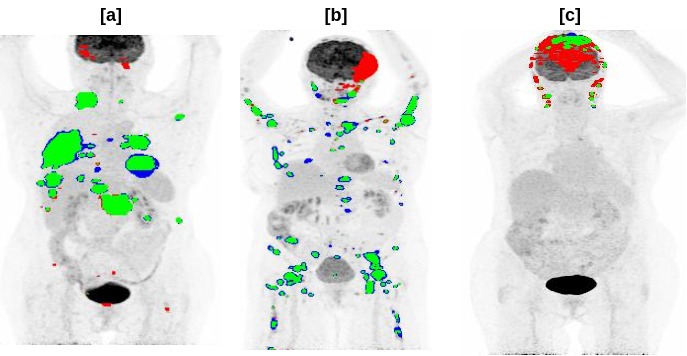


**Figure S3.** Illustration of some of the outlier cases where both the models (baseline and proposed) failed to segment the lesions that are present close to the physiologically high uptake regions such as brain, bladder, heart. [a] Lesions present near the brain and bladder are completely missed, [b] Lesions present near brain is completely missed, [c] Lesions present in the brain is partially missed. TPs (True positives) are shown in green, FNs (false negatives) in red, FPs (false positives) in blue. Physiological high uptake regions correspond to dark intensities in the images, such as brain, bladder, heart.
